# Phylodynamics and molecular epidemiology of the respiratory syncytial virus fusion protein in China

**DOI:** 10.1128/spectrum.02983-25

**Published:** 2026-05-01

**Authors:** Muhammad Nabeel Amjad, Jing Wang, Bei Shen, Huiting Xu, Muhammad Awais Ashraf, Muhammad Asif Raza, Ghayyas ud Din, Lingdie Chen, Ahsan Ali Bhutto, Lihuan Yue, Hammad ul Hussan, Dan Qian, Wei Dong, Huajie Yan, Yihong Hu

**Affiliations:** 1 CAS Key Laboratory of Molecular Virology & Immunology, Institutional Center for Shared Technologies and Facilities, Pathogen Discovery and Big Data Platform, Shanghai Institute of Immunity and Infection, Chinese Academy of Sciences 85402 , Shanghai, China; 2 University of Chinese Academy of Sciences 74519, Beijing, China; 3 Pediatric Department, Nanxiang Branch of Ruijin Hospital, Shanghai, China; 4 National Engineering Research Center for Satellite Remote Sensing Applications, Aerospace Information Research Institute, Chinese Academy of Sciences 560203 , Beijing, China; Children's National Hospital, George Washington University, Washington, DC, USA

**Keywords:** fusion protein, phylogenetic analysis, mutational analysis, evolution, drug resistance, Respiratory syncytial virus

## Abstract

Respiratory syncytial virus (RSV) infections have a high prevalence in young children, immunocompromised adults, and elderly, raising global concerns. Global RSV surveillance infers an 8%–27% mortality rate in preterm-born children, with 2.8 million/year. Continuous surveillance studies, coupled with molecular epidemiological investigations, are essential to comprehend the virus’s evolutionary dynamics and devise effective preventive strategies. The study investigates the evolutionary dynamics and molecular characterization of the RSV F-protein using bioinformatic pipelines on NCBI and GISAID data sets and molecular analysis of clinical specimens collected from children. We found S255N/G, N262S, N268I, K272M/N, and S275F/A mutations in the heptad repeat region “A” that were associated with inducing resistance toward palivizumab from bioinformatics analysis of surveillance data sets. Molecular characterization of RSV F protein from clinical specimens found L45F mutations responsible for forming new clades, L172Q and S173L substitutions for suptavumab resistance, and N276S mutations for potentially impacting palivizumab resistance. Six N-glycosylation sites are found at 27, 70, 116, 120, 126, and 500 in all RSV strains. Purifying selection, maintaining fusion protein stability, was observed. Phylogenetic analysis reveals genetic variability, with RSV B showing higher diversity than RSV A, forming distinctive clades belonging to B.D (BA9) and A.D (ON1) strains of RSV B and A, respectively. The phylodynamics of RSV indicate a uniform increase in effective population size. Understanding the F protein’s structure and dynamics is essential for elucidating the virus’s pathogenic mechanisms and developing effective vaccines and antiviral therapies.

**IMPORTANCE:**

Respiratory syncytial virus remains a major cause of severe respiratory disease in infants, the elderly, and immunocompromised populations worldwide. Despite recent advances in monoclonal antibodies and vaccines, the virus continues to evolve, posing challenges for long-term control. The RSV fusion (F) protein is central to viral entry and the primary target for neutralizing antibodies, yet little is known about its global evolutionary dynamics and drug-resistance associated changes. By integrating large-scale surveillance data with clinical isolates, our study identifies critical mutations, glycosylation patterns, and evolutionary pressures that shape the diversity of the F protein. These findings provide mechanistic insight into how RSV adapts under immune and therapeutic pressure, highlighting both vulnerabilities and conserved features of the F protein. Continuous monitoring of these evolutionary patterns will be crucial for maintaining vaccine effectiveness and informing the development of next-generation therapeutics to reduce RSV-associated morbidity and mortality.

## INTRODUCTION

Respiratory infections in humans are frequently associated with various viral pathogens, including paramyxoviruses, influenza viruses, rhinoviruses, and coronaviruses (1, 2), primarily targeting the epithelial cells of the mucosal membrane of the respiratory tract, leading to similar clinical manifestations characterized by rhinorrhea, tachypnea, wheezing, and cough (3, 4). The discovery of respiratory syncytial virus (RSV) dates back to 1955 when it was first found in chimpanzees with coryza (5), followed by RSV infections in two children in 1957 (6).

Furthermore, para-influenza and RSV exhibit clinical, pathological, and structural similarities. Classified under the Mononegavirales order and *Pneumoviridae* family, RSV possesses a single-stranded RNA bearing a negative-sense linear and non-segmented genome. It displays round and kidney shapes ranging between 150 and 250 nm and filamentous growths up to 10 µm (7). The genome size of RSV is 15.2 kb, containing 10 protein-encoding genes that encode for 11 proteins. These include structural proteins, fusion protein, glycoprotein G, small hydrophobic proteins, and three non-structural proteins, NS1, NS2, and M2-2 (8). The RSV helix comprises a 12–15 nm diameter. The enveloped pleomorphic structure of RSV has a nucleocapsid core wrapped with a lipid layer. RSV is a notorious pathogen associated with infant hospitalization due to acute respiratory tract infections (RTIs). Airway diameter and airway edema are major determinants of disease severity in this age group (9). Particularly, preterm-born infants are at higher risk of infection due to their compromised immunity. Global RSV surveillance infers an 8%–27% mortality rate in preterm-born children under 5 years of age (10). RSV hospitalization rate varies with the age of children as 26.3/1,000 for under 6 months, 11.3/1,000 for 6–11 months, and 1.4/1,000 for children aged above 12 months, causing 2.8 million cases and 118,000 deaths in 2015 (11). Recent studies have reported an estimated 74,000 annual deaths associated with RSV infections, with a mortality rate of 1.6% among adults in high-income countries, underscoring the significant morbidity and mortality burden imposed by this pathogen (12, 13).

**Fig 1:**
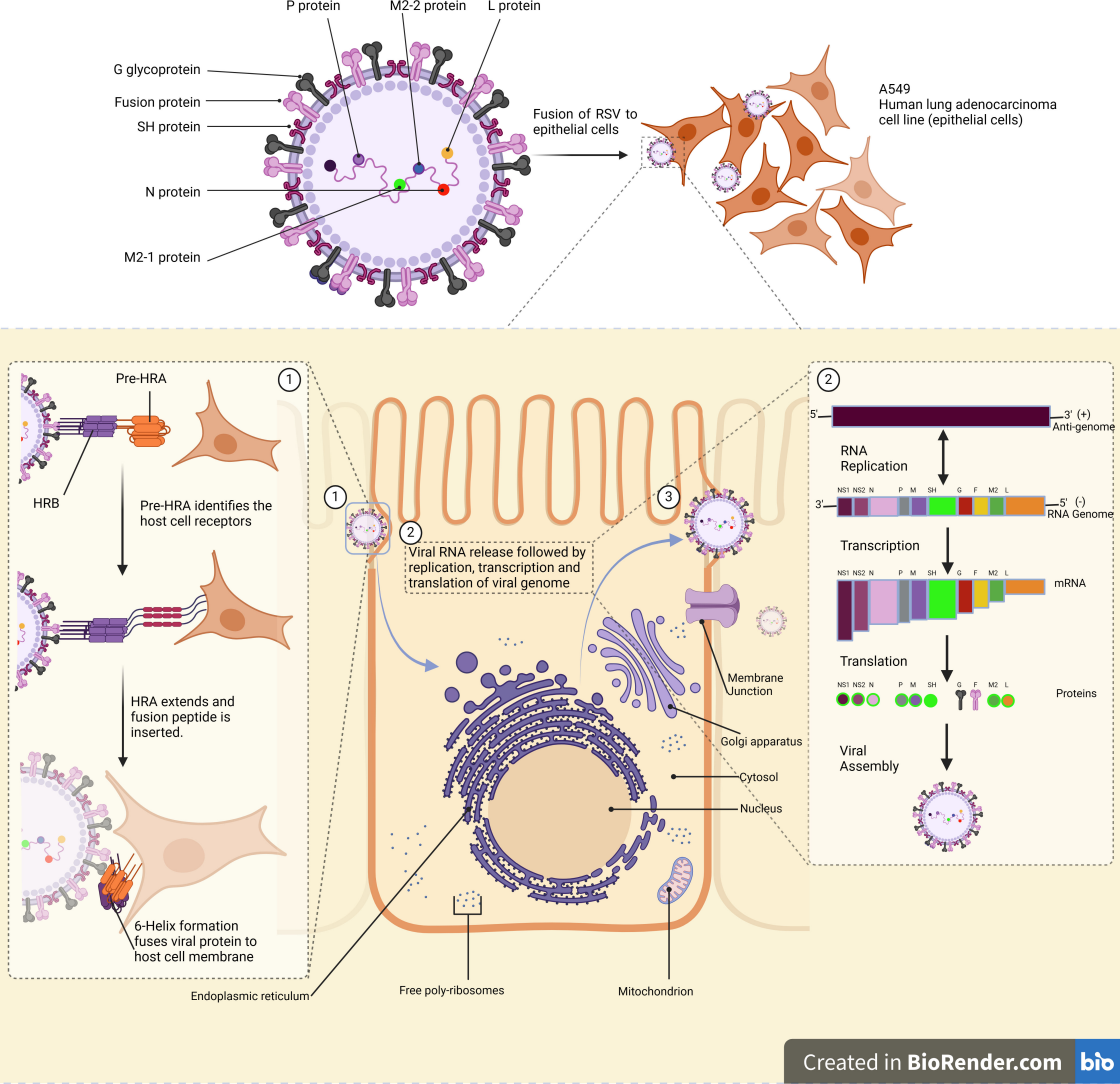
Fusion protein-mediated membrane fusion and replication of RSV. Note: RSV contains 11 structural and non-structural proteins with significant functions. Fusion glycoprotein mediates the fusion of the viral entry into the host cells. (1) In the F1 protein’s pre-fusion form, the fusion peptide (FP) precedes four short a-helices linked by three non-helical peptides. These peptides transform into a-helices upon activation, forming a long HRA a-helix that propels the FP into the target cell membrane. The HRA a-helices then trimerize, and the molecule folds, allowing the HRB a-helices to slot into the HRA grooves and form a stable 6-helix bundle (6HB). This process facilitates the fusion of the virion and cell membranes. (2) Uncoating of genomic RNA occurs after entry and release of the virus, leading to replication, transcription, and translation. Translation of G, SH, and F proteins occurs at the plasma membrane. Other proteins are transported to the cell surface via free ribosomes. (3) Viral filaments are formed after RSV assembly, leading to membrane fusion, which cleaves the cell membrane, disseminating viral particles. The image was created in BioRender.

The fusion protein of hRSV is responsible for the binding and fusion of viral particles with host target cells (Fig. 1) (14, 15). It shares similarities with other paramyxoviruses in terms of structural motifs. However, the F protein’s complete coding region (CDS) shows minimal relatedness toward other paramyxoviruses. The fusion glycoprotein, a type I glycoprotein, consists of 574 amino acids when synthesized in an inactivated F0 precursor. The precursor contains 5 to 6 N-linked glycosylation sites, which vary depending upon the type of sub-strain, and the cytoplasmic domain is palmitoylated at a cysteine (16, 17). Fusion glycoprotein shares more than 90% sequence similarity between RSV A and B strains. The variability of the fusion protein is mainly found between amino acids 62–69 and 196–210, which constitute antigenic site Ø (18) (Fig. 1).

The F0 precursor contains 15 scattered cysteine residues, which are essential for proper folding and transport. Mutations in any of these cysteines (positions 37, 313, 322, 333, 343, 358, 367, 393, 416, and 439) prevent surface expression, hindering viral replication (19). In the endoplasmic reticulum, the F0 precursor undergoes high-mannose sugar chain modifications. A trimer composed of three F0 monomers is formed and undergoes modification by host enzymes in the Golgi complex. Furin-like protease generate F1 and F2 from the C-terminus and N-terminus, respectively. Disulfide bonds link these two polypeptides. Unlike bovine RSV and other paramyxoviruses, in which cleavage occurs once, the hRSV fusion protein undergoes two cleavages at amino acid positions 109 (site I) and 136 (site II) (20). The second cleavage excised by micropinocytosis partially cleaves the 27-amino-acid residue, p27. P27 peptide lies between amino acids 109 and 136 on the genome, containing 2-3 N-linked glycosylation sites. A mature fusion protein of hRSV contains two heptad repeat regions, HRA and HRB. X-ray crystallography revealed the structure of homotrimer, containing three a-helices HRA, in the internal core constrained by antiparallel HRB a-helices forming grooves of coiled HRA trimer (21).

The RSV F protein, a crucial determinant in mediating the fusion of the virus to host cells, undergoes proteolytic cleavage to activate its function. This activation results in the formation of a metastable prefusion conformation essential for infection (22). Notably, the genetic diversity of the RSV F protein is relatively low compared to other viruses like influenza and SARS-CoV-2, with RSV A exhibiting greater genetic diversity than RSV B (23, 24). Mutations within the F protein, particularly in major antigenic sites, have been implicated in the development of resistance to neutralizing antibodies such as palivizumab, which targets a specific epitope on the RSV F protein (24, 25). In addition to neutralizing antibodies, the evolution of the RSV F protein has implications for vaccine development. Recent studies have identified alterations in the outer surface of the F protein, suggesting ongoing genomic changes that may enable the virus to evade vaccine-induced immunity (26). Overall, the evolutionary trajectory of the RSV fusion protein reflects continuous adaptation, underlining the importance of ongoing surveillance and research to inform effective therapeutic and preventive measures against RSV infections. Due to the conserved nature of the fusion protein, its role in viral fusion and disease progression, and the potential neutralization effect of antigenic sites, the fusion protein is an ideal target candidate for neutralizing antibodies and vaccines. Hence, this study examined the molecular evolutionary patterns and potential resistance-associated mutations in the F protein CDS.

## MATERIALS AND METHODS

### Bioinformatics analysis of RSV

#### Sequence retrieval

We fetched global surveillance sequence data for both subtypes of RSV (A & B) from the NCBI Virus and GISAID databases. The collection dates for specimens were set to 01/01/2013 to 31/12/2022 due to the availability of clinical specimens from the relevant era for comparative analysis. Sequences grouped under hRSV A taxid: 208893, hRSV A taxid: 11259, and hRSV B taxid: 208895 were downloaded. A local FASTA-format data set containing genomes from both NCBI and GISAID included 16015 RSV A and 11318 RSV B sequences. Metadata was also extracted for respective sequences.

#### Reference genomes

Human respiratory syncytial virus (Accession number: M74568) (27) and human respiratory syncytial virus 9320 (Accession number: AY353550) (28) were selected as reference genomes for RSV A and B, respectively. These strains were used for splicing, mutational, and phylogenetic analyses. A bovine orthopneumovirus isolate, bovine respiratory syncytial virus (bRSV) strain (Accession number: OM860285) isolated from Italy, was used as a root domain of both RSV A & B phylogenetic tree as an outlier (29, 30).

#### Bioinformatics pipelines for genomic analysis

We used MAFFT specific region extraction tool LAST (https://gitlab.com/mcfrith/last) to find, extract, and align the specific region from the database using reference sequences for each protein (31). The minimum coverage was ≅ 0.99%, undertaking ±10 base pairs of change as an acceptable range. CD-hit removed redundant sequences with a threshold of 0.9 similarity.

#### Phylogenetic analysis

The current study used the Generalized Time Reversible (GTR) model for nucleotide-based data sets. Phylogenetic trees generated by FastTree 2.1 were subjected to FigTree to create an interactive interface for the trees. Heuristic algorithms are used in FastTree structure phylogenetic trees according to evolutionary relationships inferred from the nucleotide sequences using the GTR model (32). Re-root the phylogenetic tree at the bRSV strain (Accession number: OM860285). Major clades were highlighted, and branches were colored based on their year of isolation.

#### BEAST analysis

BEAST v2.7.5 was used to estimate the time-based evolutionary hypothesis with multiple tree topologies relying on the Markov Chain Monte Carlo (MCMC) method (33). The model used a relaxed/random molecular clock as the viruses have a higher rate of evolution. The mean clock rate was set at 7.9E-4. The site model for the study has a gamma category count of 4, along with the GTR model. A coalescent Bayesian skyline with a chain length of 100 million generations and 10% burn-in standard was set for the study. The exported XML file was used in BEAST v2.7.5 to conduct the analysis. Tracer v1.7.2 was used for phylogenetic inference and visualizations.

#### Geographical mapping

ArcGIS Pro was used to visualize the spatial data of RSV surveillance using the number of sequences recorded from each country during the study period. Frequency maps were generated using color gradients to indicate the incidence rate in each participating country between 2013 and 2022.

### Molecular and computational analysis of clinical samples

#### Sample collection

In all, 230 Nasopharyngeal swabs were collected from patients who visited the Pediatric Department, Nanxiang Branch of Ruijin Hospital, Shanghai, China, between 2009 and 2022. Samples were collected using sterile nasal swabs and immediately transferred to viral transport media. Each sample was assigned a unique identifier.

#### Amplification of fusion protein

**Fig 2:**
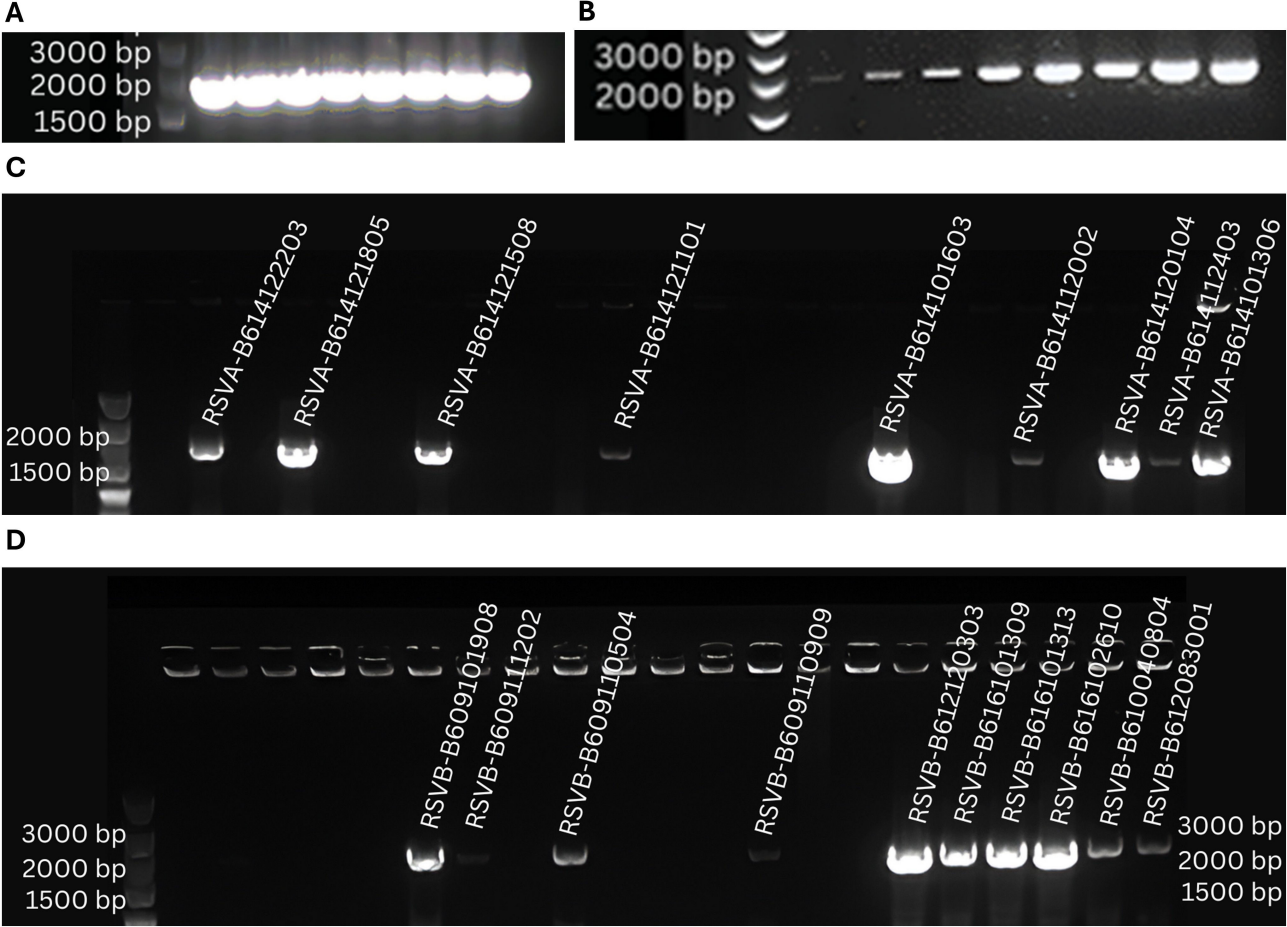
Gel electrophoresis blots from RSV primer optimizations and F protein amplification. Note: (**A**) The gel electrophoresis image contains the amplification bands of ATCC VR 1540 strain for RSV A using the RSVA/P/1770 primers at 52.7°. The product size of the respective primers is 1,770 base pairs. (**B**) This gel electrophoresis blot reflects amplified F protein of ATCC VR 1400 strain for RSV B using the RSVB/D/2028 primers with a product size of 2,028 base pairs. The image indicates the effect of increased annealing temperature in a gradient PCR system, ranging from 51° to 58° with a rate change of 1°. (**C**) The amplified F protein with approximately 1,770 bps from clinical specimens of RSV A and (**D**) reflects the amplified F protein of RSV B clinical isolates with 2,028 bps size.

Using the manufacturer’s centrifugation protocol, a HiPure RNA extraction kit (Magen, R4171-03) was used to extract RNA from nasal specimens. Extracted viral RNA was used for Multiplex qRT-PCR using multiple primer sets and probes labeled with different fluorescent dyes in a single reaction to detect multiple RNA target sequences. Positive specimens from qRT-PCR were reverse transcribed to generate complementary DNA of viral RNA using YEASEN Hifair AdvanceFast 1st strand cDNA synthesis kit. cDNA transcribed from viral RNA was subjected to standard PCR amplification using the TOYOBO KOD FX kit. Multiple amplification primer sets were designed using Primerpremier 5 (www.premierbiosoft.com) and SnapGene (www.snapgene.com) software (Table 1). The RSVA/P/1770 primer set was used for RSV A fusion protein amplification. The conditions for RSV A samples begin with pre-denaturation at 94°C for 5 min, followed by 40–45 cycles of 94°C for 30 s (denaturation), annealing at 52.7°C for 30 s, and extension time of 1 min 30 s at 72°C. The final extension time was 7–10 min at 72°C (Fig. 2A). The RSVB/D/2028 primer set was used for the amplification of the RSV B fusion protein. The RSVB/D/2028 primer set has pre-denaturation at 96°C for 5 min. The amplification cycles (40–45 repeats) began with denaturation at 98°C for 10 s, followed by annealing at 58°C for 30 s and an extension of 2 min at 72°C. The final extension was 10 min at 72°C before the program was held for 4°C (Fig. 2B). Gel electrophoresis using 1% agarose gel was used to examine the amplified product (Fig. 2C and D). Vazyme DL 5000 marker was used as a DNA ladder. According to the manufacturer’s protocol, bands corresponding to 1,770 bps for RSV A and 2,028 bps for RSV B were purified using StarPrep DNA Gel Extraction Kit (GenStar, D205-04).

**TABLE 1:** List of amplification primers^*a*^

No.	Primer sequence	Strain/ID	Product size
1	GGGGCAAATAACAATGGAGTT	RSVA/P/1770/F	1,770 bp
2	CATTGTAAGAACATGATTAGGTGCT	RSVA/P/1770/R	
3	ATGATTTTCACTTTGAAGTGTTC	RSVA/M/2509/F	2,509 bp
4	TGTCTTACAAGCAGTGCATG	RSVA/M/2509/R	
5	CAATCCAACCTGCTGGGCTAT	RSVA/D/2336/F	2,336 bp
6	CCTTCGTGACATATTTGCCCC	RSVA/D/2336/R	
7	TAAGCAGGGCAAATAACAATGGAGTTGCT	RSVA/DC/1935/F	1,935 bp
8	TAAGCAACAGATGGTAAGTTAATCTGGCA	RSVA/DC/1935/R	
9	CCACAAACAAAAGAGACCCA	RSVB/D/2028/F	2,028 bp
10	GCTTAGTGTAACTGGTGTGT	RSVB/D/2028/R	
11	TAAGCACCACAAACAAAAGAGACCCA	RSVB/DC/2028/F	2,028 bp
12	TAAGCAGCTTAGTGTAACTGGTGTGT	RSVB/DC/2028/R	
13	GTATATGTGGCAACAATCAACTTTGC	RSVB/M/2514/F	2,514 bp
14	TGTTTAACATGAAGTTTTGCCTCACTAG	RSVB/M/2514/R	
15	CGAAAACACACCACTCCACAC	RSVB/P/1900/F	1,900 bp
16	GTGGTTTTTTGTCTATTTGCTG	RSVB/P/1900/R	

^*a*^ This table contains the designed primers to specifically amplify the fusion protein of RSV A and B strains. The forward primers start from the G glycoprotein portion and end at Matrix protein M2 covering the whole fusion protein within the viral genome.

#### Sequencing and phylogenetic analysis

“Chain termination method” or the Sanger sequencing to determine the sequence of nucleotide bases in DNA was used in this study to obtain sequences from amplified clinical specimens. Multiple amplification and short amplicon primer sets were used for RSV A & B to overcome limitations of Sanger sequencing technology, details of which are mentioned (Table 2). The data were cleaned and curated based on the reliability and accuracy of cladograms. We designed a phylogenetic generalized time-reversible tree model based on nucleotide sequences obtained from the clinical specimens. FastTree 2.1 and FigTree software were used for phylogenetic analysis. Nextclade 3.17.0 was used to employ modern taxonomic categorization for clinical isolates (34).

**TABLE 2:** List of short amplicon primers^*a*^

No.	Sequence	ID	Strain	Type
1	CACTGGTATACCAACCAGTT	RSV A R2	RSV A	Reverse
2	GAGGAAACGAAGATTTCTGG	RSV B F2	RSV B	Forward
3	AGTGTTTCCTTCTGATGAGTTTGATGC	RSV B F3	RSV B	Forward

^*a*^ This table has the list of sequencing primers used in this study. These short amplicon primers were used in addition to the amplification primers used to amplify the product. Two amplification and one short amplicon primer were used for RSV A fusion protein sequencing, while two amplification and two short amplicon primers were used for RSV B.

#### Mutational analysis

As the current study emphasizes the mutations involved in drug resistance, selective antigenic sites were examined to observe substitution changes. These sites include site I from 380 to 400 amino acids, site II with 254–277 amino acids, site IV with 422–438 amino acids, and p27 site ranges between 109 and 136 amino acids; the site ∅ contains two residues, 62–69 and 196–200 amino acids. Site α2α3 β2β3 (AM14), having amino acids from 148 to 194 positions, and site MPE8, with two amino acid residues as 44–50 and 302–310, were also part of this analysis (35, 36).

#### Selection pressure

Positive and negative selection pressure for the clinical sequences was examined using the Data Monkey (https://datamonkey.org/) server, and Single-Likelihood Ancestor Counting (SLAC) and Fixed Effects Likelihood (FEL) analysis were performed to examine selection pressure on each site of genomes. Examining a better fit by permitting multiple instantaneous substitutions from RSV A & B clinical data sets was also accessed from data-monkey.

#### Glycosylation site analysis

NetNGlyc-1.0 server (https://services.healthtech.dtu.dk/services/NetNGlyc-1.0/) analyzed N-glycosylation sites from the clinical database. O-glycosylation sites were identified from NetOGlyc-4.0 (https://services.healthtech.dtu.dk/services/NetOGlyc-4.0/) server. The Technical University of Denmark-DTU Department of Health Technology designed these platforms.

## RESULTS

### Bioinformatics analysis of RSV

#### Phylogenetic analysis

**Fig 3:**
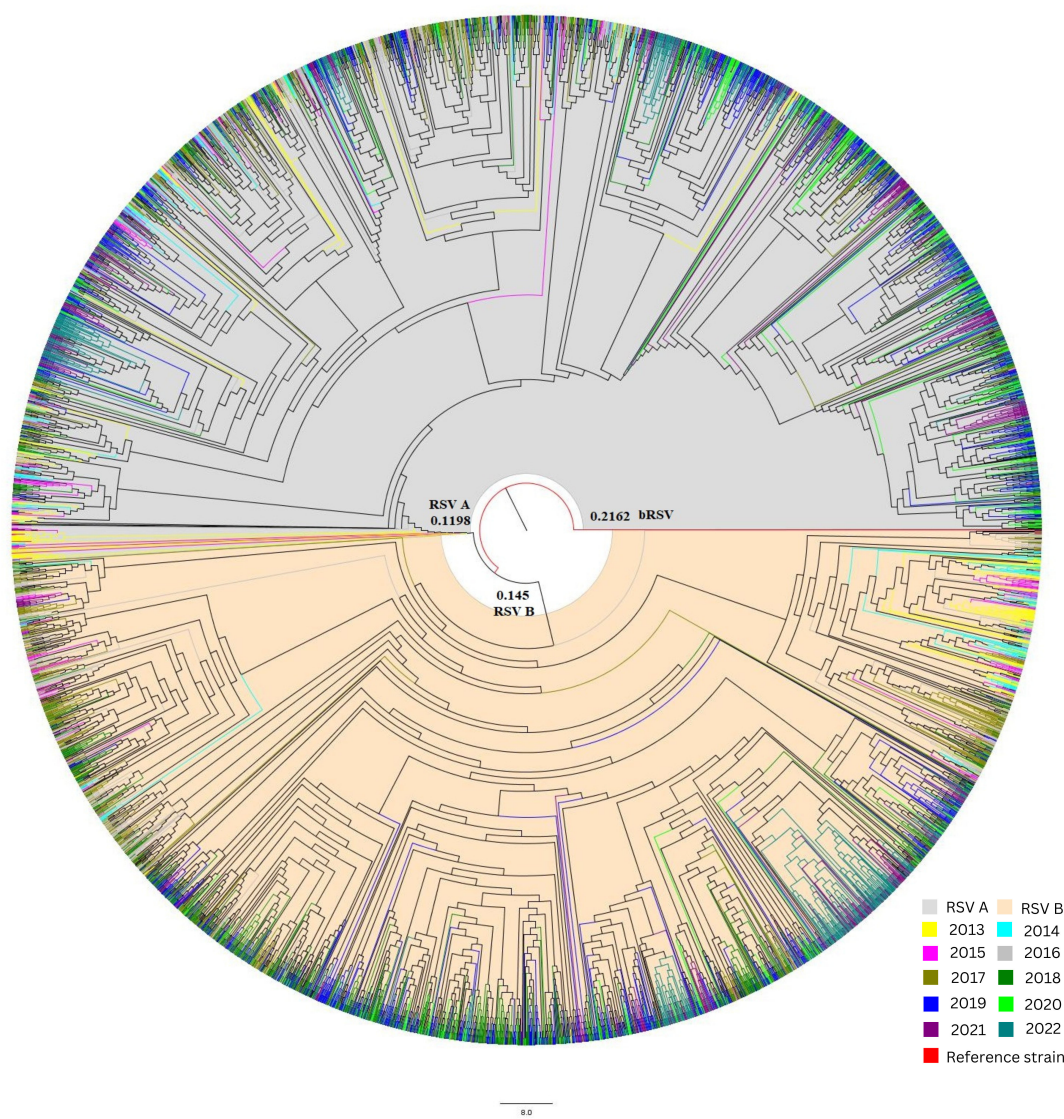
Phylogenetic tree of RSV. Note: The combined phylogenetic tree of RSV strains has a root domain of bovine RSV. The branch length of RSV A was recorded as 0.1198, and for RSV B, 0.145 from the root of the tree. The tree branches were colored based on their year of collection, ranging from 2013 to 2022. 8.0 overall distance indicates high nucleotide diversity.

Multiple sequence alignment (MSA) of the RSV A database included 2,881 sequences, while the MSA database of RSV B has 2,773 F protein CDS of 1,725 base pairs. The sequence distribution for each year exhibited significant variability (Fig. S1 is available at https://github.com/amjadnabeel/pdc_rsvf). The RSV A & B maximum likelihood (ML) tree was generated using FastTree 2.1. The current ML tree indicated a distance of 0.1198 for RSV A, while RSV B has a 0.145 distance from the root domain, indicating two sister clades formed from the bRSV strain. Year-wise separation of sequences shows clustering of multiple years in one clade. The maximum cluster distance between sister clades observed in RSV A was 0.0242 for sequences from 2013, and the rest of the database has a 0.0118 value. The evolution follows downwards with an observed distance of less than 0.0099 throughout the RSV A evolution. In the RSV B ML tree, only one major clade was observed with a distance value of 0.0199. In contrast, most sister clades were 0.0005 distance apart, indicating slow and continuous evolution (Fig. 3).

#### Mutational analysis

**Fig 4:**
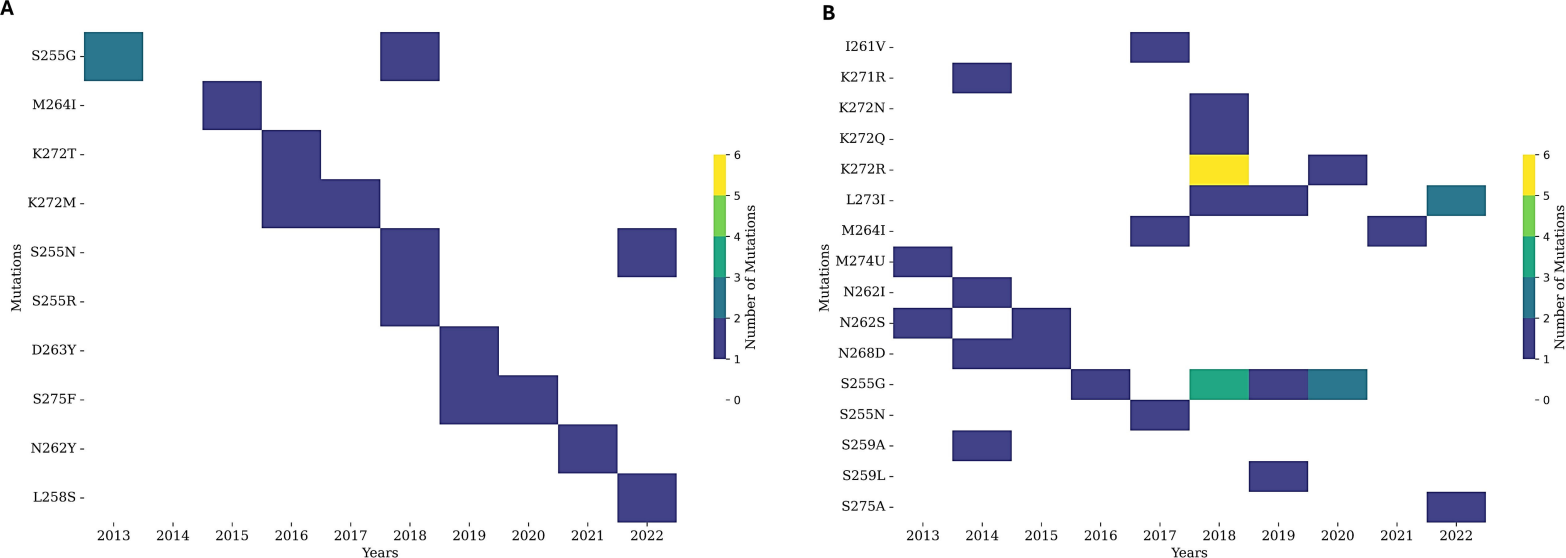
Mutations in the HRA region of RSV A & B. Note: Mutation mapping of the HRA region of RSV indicates a lower mutation count in RSV A (**A**) than in RSV B (**B**). (**A**) No mutation was observed at any amino acid residue in the HRA region during 2014. (**B**) A relatively higher number of reoccurring mutations was observed in the HRA region of RSV B.

Mutational analysis across years indicates that few mutations occurred repeatedly in the HRA region. Otherwise, no particular trend was identified in the HRA region. In RSV A, only the S255G mutation was observed in two sequences in 2013, which was later observed in one sequence in 2018, yet S255N and S255R single substitutions were also observed in 2018. No mutation was observed in sequences from 2014 in the HRA region. One mutation, M264I, was identified in 2015, while K272M was detected in 2016 and 2017, along with K272T, which was also observed in 2016. Another consecutive 2-year mutation was seen at the 275 position, changing from serine to phenylalanine. In RSV B, the most frequent mutations were recorded at 255 positions from Serine to Glycine/Asparagine. N268D, N262S/I, and L273I appeared in multiple years (Fig. 4). The highest number of mutations was recorded in sequences collected during 2018. In all, 276 amino acid positions showed a tremendous adaptation of mutation as N276S (Fig. S2A is available at https://github.com/amjadnabeel/pdc_rsvf). RSV B mutational analysis indicates a higher substitution rate than RSV A. The total number of mutations in the HRA region was higher, with certain mutations reappearing in consecutive years.

In a comparative analysis of the heptad repeat region (253–275), mutations from both strains of RSV fusion protein indicate that the target site remains highly conserved during the study period. The number of mutations increased to 13, the highest recorded in RSV B in 2018, which was reduced to 3 by the following year. Several mutations in both strains remained in a considerable range. Only 15 mutations were observed for RSV A and 35 for RSV B in the HRA region (Fig. S2B is available at https://github.com/amjadnabeel/pdc_rsvf)

#### Geographical distribution

**Fig 5:**
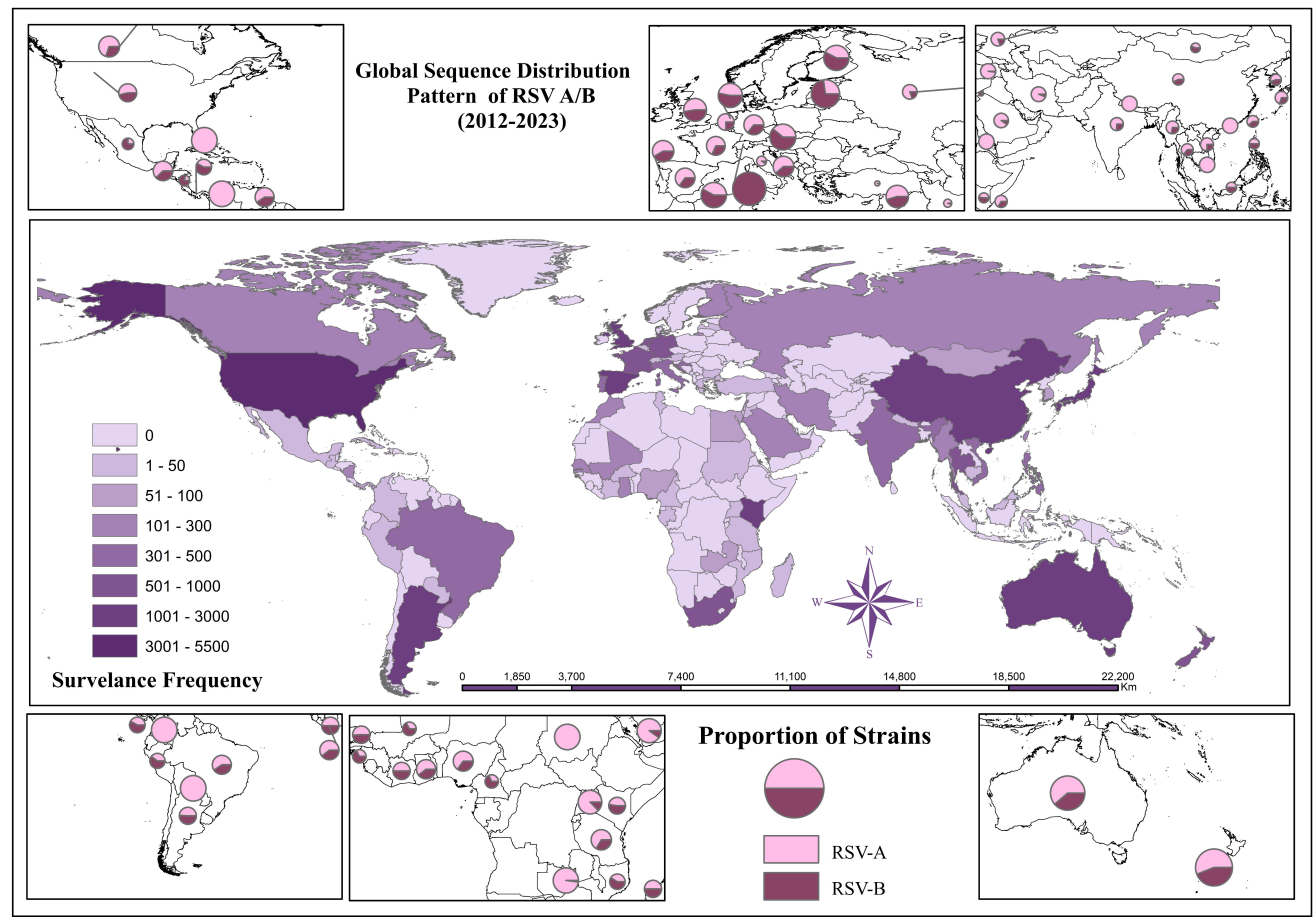
Global sequence distribution pattern of RSV during 2012–2023. Note: Geographical mapping of RSV A indicates surveillance and sequence retrievals during the study period. The frequency of the color chart indicates the United States and China as the top contributors, followed by Australia. Pie charts show the strain dominance and its burden in each region.

RSV A sequences distribution indicated most records in the United States and China, followed by Australia. Spain recorded the highest number of sequences in central Europe, while Kenya recorded the highest prevalence in Africa. The USA recorded the highest sequences for RSV B, while China, Australia, and Argentina followed sequentially in numbers. The frequency-based color distribution on the world map of geographical boundaries indicates the burden of recorded sequences across countries during the study period (Fig. 5).

#### Evolutionary analysis

**Fig 6:**
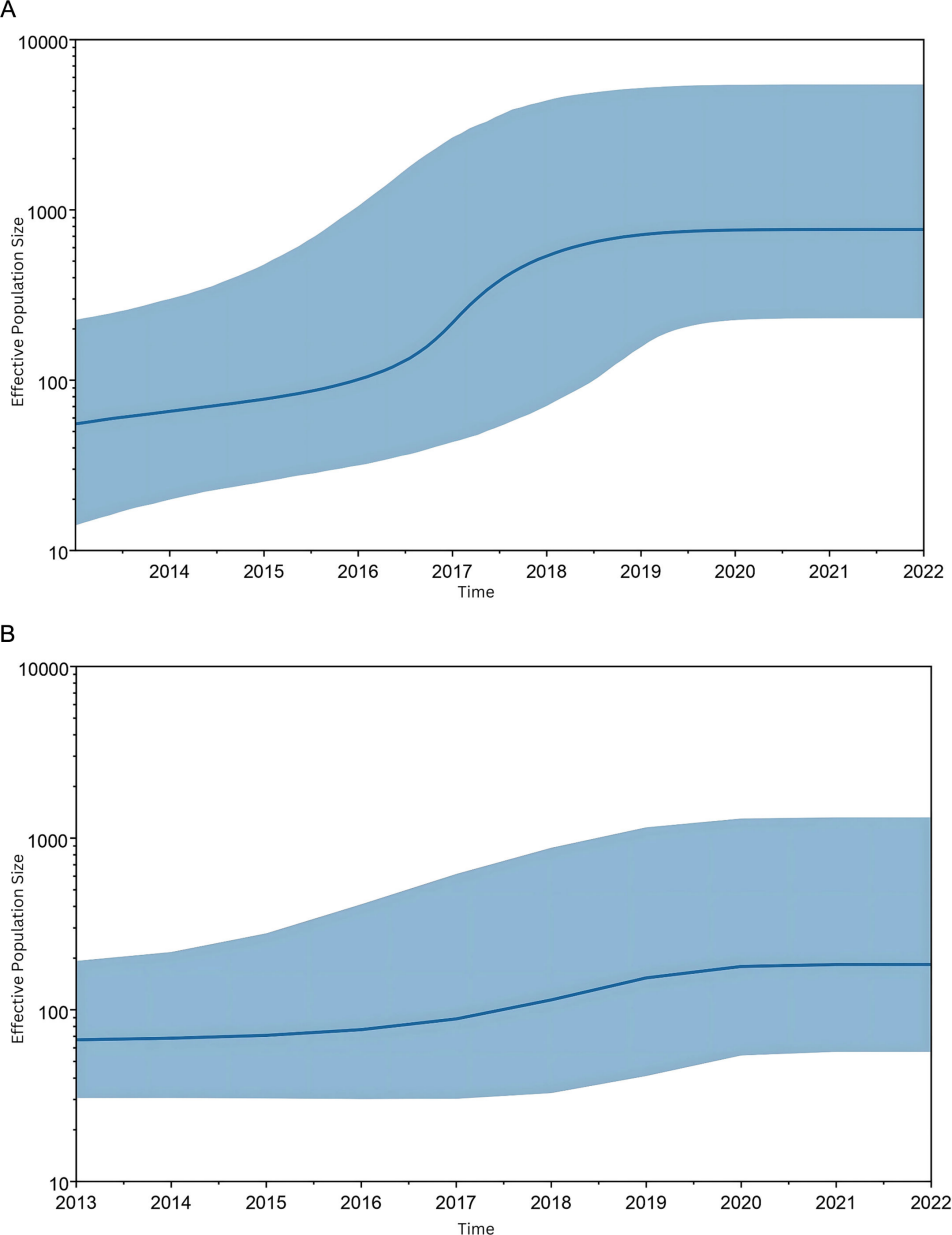
Coalescent Bayesian skyline plot of RSV (**A**). Note: BSP of RSV (**A**) indicating the change in the size of the effective population depending upon changes in nucleotide bases using the generalized time reversible model in BEAST. (**B**) Note: BSP of RSV (**B**) indicating the change in the EPS depending on changes in nucleotide bases using the GTR model in BEAST.

We used the generalized time-reversible model (GTR) as a best-fit model for predicting effective population size (EPS) to understand the evolutionary dynamics of the RSV fusion protein. Bayesian skyline plot (BSP) of RSV A with 95% Highest Posterior Density (HPD) and 10% burn-in indicates that the population size increased uniformly until 2016, when an inclined increase was observed till 2019. RSV A fusion protein evolution appeared between 2016 and 2019, while a uniform increase in EPS from 2016 to 2020 was observed in the study for RSV B. The global EPS of RSV B F protein remains lower than the EPS of RSV A from 2013 to 2022 (Fig. 6A and B).

### Molecular and computational analyses of clinical samples

#### Selection pressure

Selection pressure was applied to the clinical data sets, accessed using the Datamonkey (https://datamonkey.org/) online server. For RSV A, SLAC found zero sites influenced by positive selection, while five sites under purifying pressure were recorded. FEL recorded zero positively influenced sites but found 29 negatively influenced sites. The mean dN/dS for RSV A was −0.0264. SLAC and FEL recorded 9 and 51 sites influenced by purifying pressure/ negative pressure, respectively, and no site was influenced by positive selection. The mean dN/dS of the RSV B database was −0.047. The negative mean dN/dS for both genes indicates purifying pressure. The *P* value for both RSV A and B was ≤0.05 (Table 3).

**TABLE 3:** Selection pressure^*a*^

	Number of positive selection sites		Number of negative selection sites		Mean dN/dS	*P* value
hRSV strain	SLAC	FEL	SLAC	FEL		
hRSV A	0	0	5	29	−0.0264	≤0.05
hRSV B	0	0	9	51	−0.047	≤0.05

^*a*^ SLAC or FEL observed no positive selection sites with a *P* value of ≤0.05.

#### Glycosylation sites

N-glycosylation sites were identified using the NetNGlyc-1.0 server for RSV A and B. The server observed six common N-glycosylation sites at 27th, 70th, 116th, 120th, 126th, and 500th amino acid positions. These sites were similar in RSV A and B (Table 4). One sequence has an additional N-glycosylation site at the 566 position, while three sequences have an additional site at the 569 position. The threshold for both RSV strains was set at 0.5.

**TABLE 4:** N and O-glycosylation sites in RSV A & B^*a*^

Amino acid position	Glycosylation site	Sequence identity	Sub-group
N-glycosylation sites in RSV			
27	NITE	All clinical sequences	A
70	NGTD	All clinical sequences except RSVA-B613120904	A
70	NGTN	RSVA-B613120904	A
116	NYTL	All clinical sequences	A
120	NNTK	All clinical sequences	A
126	NVTL	All clinical sequences	A
500	NQSL	All clinical sequences	A
566	NITN	RSVA-B613123601	A
569	NITF	RSVA-B616092220, RSVA-B614112403	A
569	NRTG	RSVA-B611022801	A
27	NITE	All sequences except RSVB-B621092302	B
27	NLTE	RSVB-B621092302	B
70	NGTD	All sequences except RSVB-B621092302, RSVB-B616102610, RSVB-B616101309	B
116	NYTI	All sequences except RSVB-B616101309, RSVB-B621092302, RSVB-B616102610, RSVB-B612083001	B
116	NYTF	RSVB-B616101309	B
120	NTTK	All sequences except RSVB-B612120303, RSVB-B616102610	B
120	NPTK	RSVB-B616102610	B
126	NVSI	All the sequences except RSVB-B621092302, RSVB-B616102610, RSVB-B616101309	B
126	NVSL	RSVB-B621092302, RSVB-B616102610, RSVB-B616101309	B
500	NQSL	All the sequences	B
O-glycosylation sites in RSV			
99	Serine	RSVA-B614112403, RSVA-B612010905, RSVA-B614082103	A
118	Threonine	RSVA-B612010905, RSVA-B614112403, RSVA-B614082103	A
128	Threonine	RSVA-B612010905, RSVA-B614112403, RSVA-B614082103	A
244	Threonine	RSVA-B614121508, RSVA-B612030608, RSVA-B616092223, RSVA-B614121101, RSVA-B614121101, RSVA-B613122601, RSVA-B614082103	A
128	Serine	RSVB-B609111202	B

^*a*^ N-glycosylation and O-glycosylation sites of RSV A and B clinical samples using NetNGlyc 1.0 server on 23rd January 2024 with a threshold of 0.5 and NetOGlyc 4.0 server on 24th January 2024 with a threshold of 0.5, respectively.

O-glycosylation sites were identified using the NetOGlyc 4.0 online server. Four O-linked glycosylation sites were observed in RSV A sequences. These sites include 99S, 118T, 128T, and 244T positions, where S represents serine, and T represents threonine. Only one sequence indicated an O-glycosylation site at a 128-serine position in the RSV B data set. The threshold set for both strains of RSV was set at 0.5 (Table 4).

#### Mutational analysis of clinical samples

The clinical data sets of RSV A (33 sequences) and B (14 sequences) strains were analyzed to identify point mutations in F-protein CDS. Seven different antigenic sites were selected for mutational analysis based on their activity in the attachment of neutralizing antibodies and vaccine candidates against RSV (14). These sites have similar locations for both strains of RSV (Table 5). The absolute conservancy of cysteine residues at different coding region positions often indicates the integrity of fusion protein sequences. We found that all the 15-cysteine residues were conserved in all the strains of RSV (Fig. S3 is available at https://github.com/amjadnabeel/pdc_rsvf)

**TABLE 5:** Mutational analysis in clinical specimens^*a*^

Antigenic site	Amino acids	hRSV A			hRSV B		
		AA change	Sample identity	Percentage	AA change	Sample identity	Percentage
I	380–400	V384I	In all sequences	100%	N380S	In all sequences	100%
					V384T	In all sequences	100%
					P389S	In all sequences	100%
II	254–277	T267P	B616092220	3%	D263V	B609111202	7%
		N276S	In all sequences	100%	N276S	In all sequences	100%
IV	422–438				K433R, T434H	B616102610	7%
					S436Y	B609101908	7%
					G438A	B612083001	7%
p27	109–136	A122T	In all sequences	100%	L111A	In all sequences (except: B610040804)	92%
		K124N	In all sequences	100%	L111S, N116I, T122S, T125P, S130F	B610040804	7%
					R113Q	In all sequences	100%
					F114Y	In all sequences	100%
					L119I	In all sequences (except: B616101309)	92%
					R109K, L119F	B616101309	7%
					N121T	In all sequences (except: B616102610)	92%
					N121P, K131R, K124A	B616102610	7%
					K124N	In all sequences (except: B616102610, B612120303)	85%
					E110K, K131R, R136G	B616101313	7%
					K124H, N120T, T122I, K123I, V127L	B612120303	7%
					R109K, M115I, T118P	B612083001	7%
					T125L	In all sequences (except: B610040804, B621092302, B609110504)	78%
					T125P N116D	B621092302	7%
					T128S	In all sequences	100%
					T125P, R133M, R135L	B609110504	7%
					L129I	In all sequences (except: B621092302, B616101313, B616102610)	78%
∅	62-69, 196-210	D200G	B616092223	3%	N631	B621092302, B616112612	14%
					I64V	B616112612	
					N67T	In all sequences	100%
					K68N	B621092302, B612083001, B616120711	21%
					D200N	In all sequences	100%
					K201N	In all sequences	100%
					I206M, K209R	B621092302	7%
					K209Q	In all sequences (except: B609111608, B609101908)	78%
α2α3β3β4 (AM14)	148-194	V152I	In all sequences	100%	I148K	B616120711	7%
		L178V	In all sequences	100%	I148V, A149T	B609110504	7%
		T174P, L181F, S182F	B616092223	3%	E163R, V164M	B616101309	7%
					L172Q	B612083001, B612120303, B616101309, B616101313, B616102610, B606112612, B616112612, B616120711, B621092302	63%
					S173L	B616101309, B616101313, B616102610, B606112612, B616112612, B616120711, B621092302	49%
					S169N	In all sequences	100%
					N183M	B616102610	7%
					K191R	B621092302	7%
MPE8	44–50, 305–310				L45F	B609111608, B609101908, B609110909	21%
					S46R	B621092302, B616120711, B616112612, B616102610, B616101309, B612120303, B612083001	50%
					A47V	B616120711, B616112612	14%
					R49K	B621092302, B616102610, B616101309	21%
					L305I	In all sequences	100%

^*a*^ These mutations are based on the reference genomes from both RSV A & B. The reference strain for RSV A was M74568 and the ATCC VR-26 strain (Accession number: AY911262) for RSV B.

#### Phylogenetic analysis

**Fig 7:**
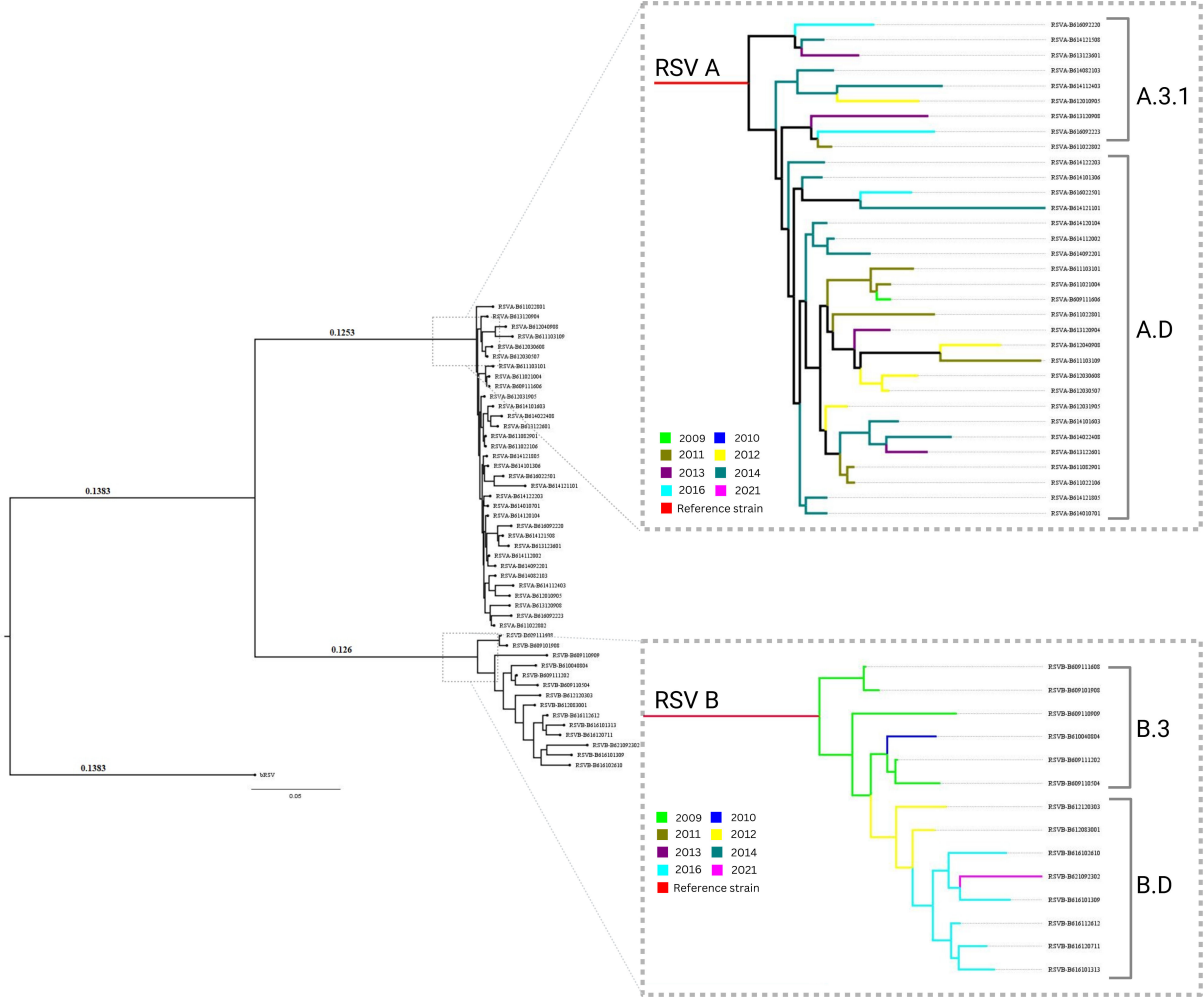
Phylogenetic tree of clinical samples. Note: The bRSV rooted phylogenetic tree forms two distinct clades, that is, RSV A and RSV B, each of which splits into subclades and branches.

Aligned data sets of both RSV strains were used for the phylogenetic analysis. We constructed the phylogenetic tree using the nucleotide-based sequences using the generalized time-reversible model in the command-line program via FastTree 2.1. The phylogenetic tree forms two distinct clades, that is, RSV A and RSV B. The branches in RSV A clades are closely related, and no major subclades were formed based on the year of isolation. These sequences belonged to A.3.1 and A.D (ON1 of classical taxonomy) clades of Nextcalde modern taxonomy. In the RSV B clade, there was a distinct subclade formation. Specimens from 2009 and 2010 were closely associated to form B.3 clade, while branches for 2012 samples were in separate junctions. 2016 samples formed a distinct subclade along with an induction of one sample from 2021 and were categorized into the B.D clade (BA 9 of classical taxonomy) (Fig. 7).

#### Multi-hit analysis

**Fig 8:**
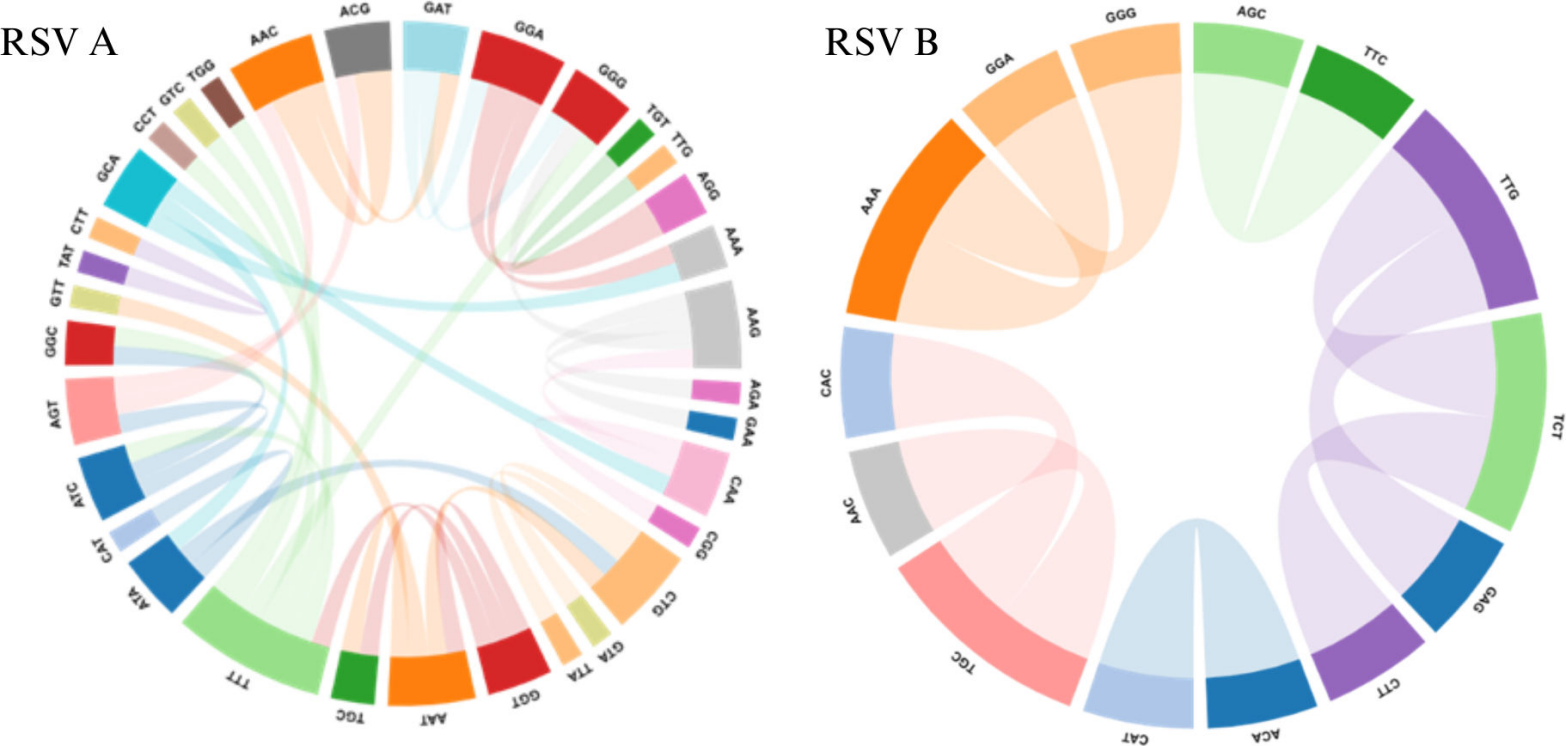
Multi-hit circus chart of RSV A & B. Note: The cirrhosis chart of codon–codon interaction networks of the RSV fusion (F) protein identified by multi-hit analysis (Datamonkey). RSV A (left) shows a dense and widespread pattern of coevolving codons, indicating stronger compensatory constraints and higher evolutionary flexibility. RSV B (right) displays fewer, more localized interactions, suggesting relative structural conservation of the F protein. (Reference strains were M74568 and AY911262).

Multi-hit statistically evaluates the impact of multiple nucleotide changes in the data set. It examines the better fit for extra-base hits by permitting multiple instantaneous substitutions in the data. The better-fit model assesses whether multiple substitutions improve the fit of the model data. In the context of RSV A, the computational model has discerned a substantial occurrence of two-nucleotide alterations. Meanwhile, for RSV B, the multi-hit model detected a relatively lower incidence of two-nucleotide changes. These changes have led to distinct amino acid variations within the viral genome. Such observations provide valuable insights into the molecular dynamics of RSV evolution and its potential impact on protein function (Fig. 8).

## DISCUSSION

RSV is an enveloped ubiquitous pneumovirus that tends to cause lower respiratory illness in children, the elderly, and immunocompromised adults. RSV affects nearly all children under 2 years of age in the United States, while causing global death tolls up to 149,000 (11, 37) and $750 million in losses in healthcare annually. In 1988, the US Food and Drug Administration (FDA) approved palivizumab, a monoclonal antibody used for high-risk patients (38), while nirsevimab was approved in 2022. Palivizumab targets the HRA region, while nirsevimab targets site Ø by acting on the discontinuous neutralizing epitopes in the pre-fusion conformation of the fusion protein (39). The FDA approved the first RSV vaccine on 3rd May 2023, and the second on 31st May 2023 (14), indicating the importance of the F protein in immunoprophylaxis of RSV infections. Multiple target regions on the F protein provide active sites for these antiviral drugs. However, the F protein is more conserved than the G protein’s hypervariable nature. Mutations in the stable and conserved nature can create adverse effects, including drug resistance. These antigenic sites include site Ø, site I, II, IV, p27, AM14, and MPE8 (35, 36).

Heptad repeat region A (HRA) overlaps with antigenic site II at positions 253–275, inducing partial to complete resistance to palivizumab due to specific mutations (20, 40, 41). S255N/G/R mutations occurred in repeated years in RSV A and B. S255N/G was the most consistent mutation in the HRA region at 764 nucleotide position due to the change of AGT to AAT bases, also observed in other studies conducted by Lin et al. (42) and Tabor et al. (43) from 2017 to 2020. However, our laboratory experimented clinical specimens found no mutation at either RSV strain’s 255 amino acid position. Other significant substitutions observed during the computational analysis of published data sets were N262S, N268I, K272M/N/Q/T, S275F/A, and K272E/Q. S275F, N262S, N268I, and K272M/N, which are affiliated with palivizumab resistance, as observed by different studies in cotton rat models (28). Mutations, including K272Q/E and S275A, were also recorded in patients treated with palvizumab (44). Similar experimental evidence was found by Qing Zhu et al.; they stated that substitutions at the 272 and 275 positions in the RSV fusion protein were resistant to palivizumab, and the K272E mutation showed resistance to motavizumab *in vitro* analysis (45).

In addition to HRA in antigenic site II, lies 276 amino acid position for which Adam et al. stated that N276S single amino acid substitution causes resistance to palivizumab (46). However, an *in vitro* experimental model shows that a single amino acid substitution at the 276 position does not induce resistance, yet is coupled with mutations at the 272 and 275 positions. N276S, in particular, reduces cell fusion (28, 47). In our clinical observation, the N276S mutation was present in all RSV specimens, along with one T267P substitution in RSV A and a D263V in RSV B. T267P and D263V mutations in site II of the F protein are not mentioned in any other studies. Post-fusion conformation of RSV F protein contains a unique antigenic site I (380–400 amino acids [36, 48]); mutations in the region may lead to altered response toward neutralizing antibodies like mAb 131-2A. Valine to isoleucine in RSV A, and threonine in RSV B at 384 AA position remained persistent in South African strains during 2019 and 2020 (49). In current clinical trials, N380S and P389S were present in all the RSV B strains, comprehended by another study conducted in China (50).

Antigenic site IV, sharing genotypic and phenotypic resemblance with human metapneumovirus, is a crucial target zone for many neutralizing antibodies. Its vitality lies in vaccine design and immune recognition. The action of 6F18 and 3M3 mAbs is resisted due to a single amino acid substitution at R429, making them arginine-dependent monoclonal antibodies (35). RB1, MK-1654, is a human parental antibody that targets antigenic site IV of RSV (51). K433 is crucial for binding ch101F mAb, whose binding is abolished by mutation by L, Q, T, and R. In the current study, we observed the K433R mutation in one RSV B strain that obliterated the binding of ch101F, according to Wu SJ et al. (52, 53).

A recent study conducted on RSV-infected children and adults by Fuentes et al. states that p27 is a dominant antigenic site (54). Vaccines consisting of the p27 sequence showed reduced viral pathology in animals, suggesting the role of p27 in virus fusion and pathogenesis (55). The frequency of alanine to threonine drift at 122 positions in the p27 region of fusion protein drastically increased from a previous study. 4.3% mutation rate changed to complete dominance in RSV A strains. The mutation rate of K124N remains the same in comparative analysis (50). In relevance to the conserved nature of p27 in RSV A, RSV B shows a huge disparity. Multiple mutations appeared predominantly throughout the target site. Substitutions in RSV B; L111A, R113Q, F114Y, L119I, N121T, K124N, T125L, T128S, and L129I occurred at a frequency of > 90%. In RSV A, GA2, and ON strains have been reported to contain such amino acid substitutions in their p27 region (36). Mutations in antigenic site ∅ affect viral pathogenicity and restrict the action of mAbs like CR9501 and vaccines against the virus. N67T, D200N, K201N, and K209Q dominant observations adhere to a previous study in scientific reports by Xiangpeng Chen et al., which have also highlighted these mutations in the Antigenic site ∅ (50). Their phenotypic influence has not been investigated yet.

RSV fusion protein contains antigenic site α2α3β3β4 on its pre-fusion conformation that is located between 148 and 194 amino acids. The significance of α2α3β3β4, also known as AM14, is the shared preserved nature in RSV and hMPV. The site tends to bind antibodies that can neutralize both viruses (56–58), suggesting a target site for broad-spectrum antiviral drugs. Mutations like S169N have also been previously reported in RSV B strains. The AM14 antigenic site is also responsible for Suptavumab binding. Our clinical analysis identified L172Q and S173L in RSV B strains. Simões et al. did *in vivo* validation of L172Q- and S173L-induced resistance against Suptavumab (23, 59). Our specimens from 2009 observed L45F substations in RSV B F protein, found predominantly in GB and SAB strains, including GB1, GB3, and GB4 (36). We observed multiple substitutions like K272Q/E, S275A, N276S, L172Q, S173L, and K433R in our specimens that have experimental verification of being drug resistance-associated mutations in the RSV fusion protein. Overall, RSV A F protein exhibits more conservation with fewer mutations, while RSV B F protein shows a higher tendency for amino acid substitutions in the genome.

Phylogenetic analysis in our study indicates that the genetic variability of the F protein varies in both strains of RSV. More pronounced genetic diversity was observed in RSV B than in RSV A. The phylogenetic tree from RSV A indicates microclustering, induced by continuous evolution of the ON1 strain of RSV A during 2012–2022 (50, 60, 61). For RSV B, distinctive clusters forecasting emerged epidemics that began with BA dominance in China during 2009 and 2010 (62). Clades formed by the 2012 and 2016 sequences belong to the BA9 substrain of RSV B (63, 64). A recent study conducted by Fudan University also highlighted the dominance of the BA9 strain in children (65).

Darwinian/positive selection is the phenomenon of nature that selects the favorable allele conferring survival advantages. It provides insight into evolution that could affect the protein’s genotypic and phenotypic functions, leading to the emergence of new strains (66). Negative selection stabilizes natural selection, where unfavorable genetic polymorphisms generated via random mutations are reversed (67). Our study did not find any positive selection site for both strains of RSV via SLAC and FEL. Furthermore, the analysis indicated that the F protein is under purifying pressure when observed at a *P* value of 0.05, also supported by several studies (49, 68–70).

Our findings include multiple N-glycosylation sites in six different locations on the genome. Leemans A et al. reported similar findings in a study indicating N27 and N70 in the F2 subunit, N116 and N126 in the p27 peptide (71), and N500 in the F1 subunit (72). In our study, another glycosylation site was observed at the N120 position, which was reported in fewer studies (73), and some strains did not have the N120 N-glycosylation site (30). O-glycosylation sites, common in post-translational modification in viral proteins, significantly influence the immunogenicity of the protein and its function (72, 74). Our study found 99S, 118T, 128T, and 244T O-glycosylation sites in a few sequences in RSV A, while only 128S was observed in one RSV B strain. Our findings are supported by a study conducted by Song J. et al. (61). Global databases have recorded RSV prevalence in high-income countries, yet surveillance data are scarce from low- to middle-income countries (LMICs). Studies conducted in LMICs indicated a high prevalence (75). Similarly, our study found that the highest surveillance was observed in the United States of America and China, followed by Europe. Few records were observed from LMICs, urging the need for global, unbiased surveillance of infectious diseases. We constructed global phylodynamics of RSV A and B genotypes using publicly available genomes of the F protein. We estimated the rate of change in the population size of RSV based on the F protein. We used a generalized time-reversible model with randomized relaxed clock modeling. No study to date has addressed the topic globally, yet a study conducted in Taiwan indicates an increase in EPS during 2005 within the country (68). Certain other studies conducted Bayesian coalescent skyline construction via BEAST on G glycoprotein (26). At the same time, some focused on the complete genomes (70), indicating the F protein is not responsible for the emergence of new strains. While our study offers valuable insights into the phylodynamics and epidemiology of RSV, it has certain limitations, including a small sample size and decade-old samples with low volume majority of which were not suitable for viral propagation.

### Conclusion

The conclusion of our study indicates that multiple substitutions were observed in the surveillance study that were responsible for inducing drug resistance in RSV. Mutations in the fusion protein’s HRA region, essential for palivizumab binding, have been observed, with S255N/G being consistent, impacting drug efficacy. Other mutations like N262S, N268I, K272M/N, and S275F/A are linked to palivizumab resistance, conformational changes in fusion protein from pre- to post-F alter antigenic sites, affecting neutralizing antibody binding. Notably, mutations in antigenic site I (384 position) affect antibody binding, influencing vaccine efficacy. Antigenic sites IV, ∅, and AM14 are targets for neutralizing antibodies and vaccine development, with mutations affecting their efficacy. Phylogenetic analysis reveals genetic variability, with RSV B showing higher diversity than RSV A. Selection pressure analysis indicates purifying selection, maintaining fusion protein stability. Glycosylation plays key role in immunogenicity, with multiple N-glycosylation sites identified, influencing protein function. Seasonality patterns vary globally, with RSV prevalence linked to climate factors. Surveillance data are crucial, with LMICs underrepresented. Phylodynamics analysis of RSV A and B genotypes using fusion protein genomes reveals population dynamics, though limited global studies. Whole-genome sequences are essential for comprehensive phylodynamic understanding. This research contributes to understanding RSV epidemiology and molecular evolution to devise preventive strategies.

### Future research directions

The project will progress to the next stage, focusing on validating computational predictions and strain identification. Clinical specimens will undergo whole-genome sequencing, and animal models will be used to investigate the impact of mutations on drug resistance against palivizumab and suptavamab. Further analysis for phenotypic characterization of glycosylation sites and their significance would be examined for the RSV fusion protein.

## Data Availability

RSV fusion protein sequences of clinical isolates were deposited to GenBank (accession number range PP647837–PP647883) and are publicly available.
